# Secreted TRAIL gene‐modified adipose‐derived stem cells exhibited potent tumor‐suppressive effect in hepatocellular carcinoma cells

**DOI:** 10.1002/iid3.372

**Published:** 2020-11-06

**Authors:** Zhuo Liu, Shaojie Li, Tiexiang Ma, Jian Zeng, Xin Zhou, Huanyu Li, Min Tang, Xiang Liu, Feng Li, Bin Jiang, Ming Zhao, Ying Chen

**Affiliations:** ^1^ Depatment of General Surgery (Three) Xiangtan Central Hospital Xiangtan Hunan China; ^2^ Department of Oncology (One) Xiangtan Central Hospital Xiangtan Hunan China

**Keywords:** adipose‐derived stem cells, apoptosis, gene modification, hepatocellular carcinoma, metastasis, migration, TNF‐related apoptosis‐inducing ligand, tumor growth

## Abstract

**Objective:**

Considering the potential of adipose‐derived stem cells (ADSCs) migrating towards cancer cells, this study was performed to explore the function of tumor necrosis factor‐related apoptosis‐inducing ligand (TRAIL) modified ADSCs on the development and progression of hepatocellular carcinoma (HCC).

**Methods:**

ADSCs were extracted from human adipose tissues and identified through immunofluorescence and flow cytometry. Oil red staining and alizarin red staining were performed to clarify the differentiation potential of ADSCs. AAV‐CMV‐sTRAIL was transfected into ADSCs before Western blot and Transwell measurements. sTRAIL‐ADSCs were cocultured with HCC cells to explore its effect on the proliferation and apoptosis of HCC cells. The possible effect of sTRAIL‐ADSCs or ADSCs on tumor growth and metastasis was determined in vivo using xenograft nude mouse models.

**Results:**

ADSCs were successfully extracted from adipose tissues, which were confirmed by cell morphology and positive expressions of CD44 and CD105. ADSCs were found with differentiation potential. After transfection, TRAIL was stably expressed in sTRAIL‐ADSCs. Both ADSCs and sTRAIL‐ADSCs can migrate towards HCC cells. In addition, sTRAIL‐ADSCs can promote the cell apoptosis and inhibit cell proliferation in vitro, on parallel it can also suppress epithelial‐mesenchymal transition, tumor growth, and metastasis in vivo.

**Conclusion:**

TRAIL modified ADSCs can migrate towards HCC cells to inhibit tumor growth and the metastasis of implanted HCC tumors, which hints TRAIL modified ADSCs may be a new therapeutic approach for HCC treatment.

## 1 INTRODUCTION

Liver cancer, only secondary to lung cancer and gastric cancer, is the third leading cause of cancer‐related death with an annual incidence of 850,000, among which hepatocellular carcinoma (HCC), a primary malignant neoplasm derived from hepatocytes, accounts for more than 90% of all cases of primary liver cancer.^1^, ^2^, ^3^ The etiology of HCC is complicated and the predominated risk factors for HCC mainly include Hepatitis B virus, Hepatitis C virus, alcohol abuse, and aflatoxin B1 exposition.^4^ Although various therapeutic strategies including curative and palliative treatment options have been proposed, the prognosis for HCC patients was far from satisfactory with a mortality rate of 95% and a 5 years survival rate of 6.9%, which may accredit to its low resectability rate and high recurrence after resection.^4^, ^5^, ^6^ Majority of HCC patients are advanced from cirrhosis, in which chronic inflammation, aside from angiogenesis, tumor microenvironment, and microenvironment, is a central process for HCC development.^7^ Therefore, more efforts are required to explore the therapeutic target for HCC to mitigate the malignant transformation.

Tumor necrosis factor (TNF)‐related apoptosis‐inducing ligand (TRAIL) belongs to the superfamily of TNF, which was first described to induce cell apoptosis in cancer cells in 1995 by interacting with death receptors DR4 and DR5.^8^ Characterized by its specific targets to cancer cells, TRAIL is perceived as a major physiologic weapon against cancer for it has no toxicity to normal cells.^9^ Nevertheless, the development of TRAIL gene therapy was obstructed by the missing of efficient and low toxic vectors.^10^ Adipose‐derived stem cells (ADSCs) are a new group of multipotent stem cells, with similar differentiation capability similar to mesenchymal stem cells.^11^ ADSCs are widely applied for their potentials to differentiate into osteocytes, adipocytes, neural cells, vascular endothelial cells, cardiomyocytes, pancreatic β‐cells, and hepatocytes. Meanwhile, ADSCs with higher yields and lower harvest site morbidity have been gradually recognized as an attractive substitute for tissue and organ transplantation.^12^ ADSCs are reported to associate with the expressions of cancer‐related cytokines, chemokines, and inflammatory biomarkers, covering from an insulin‐like growth factor, hepatocyte growth factor, transforming growth factor beta‐1, vascular endothelial growth factor, interleukin‐8, B‐cell lymphoma‐2 (Bcl‐2), and interleukin‐10.^13^ In this regards, ADSCs have been proposed as either as a vector or as an adjunct treatment approach to increase chemosensitivity and induce apoptosis of cancer cells.^14^ However, whether ADSCs can induce cell apoptosis, necrosis, or pyroptosis in cancer cells remains controversial. Researches in bladder cancer cells evidenced that the ADSC‐conditioned medium can induce cell apoptosis in a caspase‐dependent way.^15^ Exosomes derived from ADSCs are documented to facilitate HCC suppression through promoting natural killer T‐cell antitumor responses in rats,^16^ suggesting the antitumor effect of ADSCs. In contrast, coculture with ADSCs was showed to promote cell proliferation in human squamous cell carcinoma cells,^17^ as well as in human breast cancer cells.^18^ Although conditioned medium from ADSCs was reported to inhibit proliferation and enhance cell apoptosis in HCC cells,^19^ no study explored the role of modified ADSCs on the biological function of HCC cells. Therefore, this study combined the migrating potential of ADSCs towards cancer cells with TRAIL gene therapy to explore the possibility of TRAIL modified ADSCs in regulating cell proliferation and metastasis of HCC cells.

## 2 MATERIALS AND METHODS

### 2.1 Isolation and culture of ADSCs

Adult adipose tissues were obtained from healthy females (aged 25–45 years) who undergone liposuction in People's Hospital of Xiangtan City, Hunan, China. All included subjected signed the written informed consents. The adipose tissues were kept in a container which was sterilized using alcohol wipes. About 15 g tissues were extracted in asepsis condition and washed with phosphate‐buffered saline (PBS) buffer in a 10 ml culture dish to remove the red blood cells. Ophthalmic scissors was used to remove blood vessels and to cut the tissues into pieces. After that, the tissues were digested with 5 ml of 0.1% type I collagenase (Sigma‐AldrIch, Merck KGaA) for 30 min, and then the digestion was terminated by adding 10 ml of minimum Eagle's medium (MEM) culture medium (Sigma) containing 10% fetal calf serum (Gibco). The digested cells were flapped using a pipettor till no cell mass. The digestive fluid was filtered through a 200 mesh cell strainer and transferred into a centrifuge tube for centrifugation under 1500 rpm for 10 min. Then the adipose tissues and supernatant was abandoned, while the remained cells were resuspended with red blood cell lysis buffer for 1 min. The resuspension was ended and the cell lysis buffer was subjected to centrifugation at 1500 rpm for another 10 min, with the supernatant removed. Subsequently cells were resuspended with MEM culture medium containing 15% serum and then inoculated into aseptic culture flasks at the appropriated concentration for incubation at 37°C with 5% CO_2_ and certain saturation humidity. The culture medium was replaced on the 5th day, after that the culture medium was replaced every 3 days. Cell passage was performed once the primary cells reached 85% confluency. Cell morphology was observed under an optical microscope.

### 2.2 AAV‐CMV‐sTRAIL transfection

Based on method proposed by Synder and Clark,^20^ the AAV‐CMV‐sTRAIL (10^11^–10^14^/ml) was produced by Genetic Department of Harvard University Gene Therapy. The first generation of ADSCs with 90% confluency was separately inoculated into three culture flasks (25 cm^3^) with the concentration of 7.75 × 10^4^ cells/ml, each flask for 1.5 ml. The inoculated cells were incubated with MEM culture medium with 20% fetal calf serum. After 3 days of incubation, two flasks were transfected with AAV‐CMV‐sTRAIL: abandon the culture medium; diluted AAV‐CMV‐sTRAIL (based on multiplicity of infection of 50) to fully cover the cells; incubation at 37°C for 2 h; shake every 15 min to allow the AAV‐CMV‐sTRAIL to fully attach the ADSCs. After that, cells were incubated with certain amount of MEM culture medium containing fetal calf serum (final concentration of 10%) for routine adherent culture.

### 2.3 Immunofluorescence staining

The slides were coated with polylysine and maintained at an incubator overnight. Then double distilled water was used to wash the slides for twice. After cell passage, cells were inoculated in the well plates which contain slides. The cells were taken out and washed with PBS for two times once cell confluency reached 85%, followed by fixation under 95% ethanol for 15 min, PBS wash for twice, reaction with 3% H_2_O_2_ at room temperature for 15 min, PBS wash for 3 min × 2. Additionally, the slides were blocked with 5% goat serum for 1 h at room temperature before incubation with 5% goat serum prepared primary antibodies of CD44 (1:100; ab157107), CD105 (1:100; ab157107), TRAIL (1:100; ab9959; Abcam) at 4°C for 4 h. After that, slides were washed with PBS for 3 min × 3 and incubated with fluorescence labeled secondary antibody (1:200) at room temperature. Two hours later, the incubation was terminated and the slices were washed with PBS for 3 min × 2 before sealing with neutral resins. The dried slides were observed under a fluorescence microscope (Invitrogen).

### 2.4 Flow cytometry for antigen

Cells were first digested with 0.25% trypsase—EDTA (Gibco) for 3 min before MEM culture medium containing 10% serum was added to terminate the digestion. After digestion, cells were flapped for counting and transferred into a centrifuge tube for centrifugation at 1500 rpm for 10 min. The supernatant was abandoned and cells were resuspended with 200 μl of fluorescence activated cell sorte (FACS) loading buffer. Cells (1 × 10^6^) were probed with each 2 μl of antibodies of CD44 (1:100; 3570S), CD45 (1:100; 13917S; Cell Signaling Technology), CD105 (1:100; ab11414), CD34 (1:100; ab81289), CD116 (1:100; ab95684) and FITC‐CD166 (1:100; ab219139; Abcam) on ice for 30 min. After FACS loading buffer washing for two times, cells were fixed with 10% formalin for determination of positive rate of antigens.

### 2.5 Oil red O staining

The 4th generated ADSCs were cultured with MEM culture medium containing 10% serum till 80% confluency. After that cells were added with 1 μl/L dexamethasone + 0.5 μl/L IBMX + 60 μl/L indometacin + 10 μl/L insulin for incubation of 2 weeks. Once cell confluency reached 85%, cell slides were washed with PBS for twice and fixed by 4% paraformaldehyde for 15 min, followed by PBS wash for twice, 60% isopropanol rinsing, oil red O staining for 10 min and differentiation with 60% isopropanol. Then cells were washed in distill water and restained with Mayer hematoxylin before washing in tap water for 1– 3 min and in distill water. Slides were mounted using glycerogelatin.

### 2.6 Alizarin red staining

The 4th generated ADSCs were cultured with MEM culture medium containing 10% serum till 80% confluency. After that cells were added with 1 μl/L dexamethasone + 10 mmol/L β‐phosphoglycerol + 50 μl/L ascorbic acid for incubation of 2 weeks. Once cell confluency reached 85%, cell slides were washed with PBS for twice and fixed by 4% paraformaldehyde for 15 min, followed by PBS wash for twice. Then the slices were stained with 0.1% alizarin red‐Tris (pH = 8.3) for 30 min at 37°C before distill water washing and observation under an inverted microscope.

### 2.7 Transwell assay

The apical chamber was covered with 1 × 10^5^ cells/ml of LacZ labeled ADSCs, or NIH3T3, or sTRAIL‐ADSCs, while the normal liver histolysate, HCC histolysate, Huh7 culture medium, or L02 culture medium was arranged in the basolateral chamber. The Transwell coculture system was maintained at 37°C for 24 h with 5% CO_2_. After incubation, the filter membrane was taken out and washed with PBS with attached cells removed. The membrane was fixed with 0.5% glutaraldehyde and stained with X‐gal at 37°C for 24 h with 5% CO_2_. After that, blue cells were observed in five random selected high power fields (×200). The migration rate was calculated using the MEM in 10% FBS as basic control.

### 2.8 Flow cytometry for cell apoptosis

Cells were washed with PBS and digested using pancreatic enzymes before centrifugation at 1000 rpm for 5 min at room temperature. The centrifuged supernatant was removed and cells were resuspended in PBS for centrifugation at 1000 rpm for 5 min at room temperature. The supernatant was abandoned again and cells were resuspended with 490 μl of precold 1 × binding buffer (cell concentration of 10^5^–10^6^/ml) resuspended with 490 μl of precold 1 × binding buffer (cell concentration of 10^5^–10^6^/ml), in which 5 μl of AnnexinV‐FITC and 5 μl of PI were added. The resuspension was conducted at ice for incubation of 10 min. Cell apoptosis was determined using flow cytometry (FCM).

### 2.9 Cell Counting Kit‐8 assay

Cells were inoculated at a 96 well plate at the density of 2 × 10^3^ cells/well. Three duplicate wells were set for each cell. The plate was incubated at 37°C with 5% CO_2_, during which 10 μl of Cell Counting Kit‐8 (CCK‐8) solution was added in each well for further incubation of 2 h. After that, culture medium was abandoned and the plate was washed with PBS for twice. Absorbance at the wave length of 450 nm was determined based on three duplicate measurements. The cell viability curve was accordingly drawn.

### 2.10 Reverse transcription polymerase chain reaction

PPA cells were dissolved in 1 ml of Trizol (Thermo Fisher Scientific) for RNA extraction based on instruction on Trizol reagent. After quantification, the RNA was subjected to reverse transcription into complementary DNA. Fluorescence quantitative PCR kit (Takara) was used to configure the PCR system. Amplification and detection were performed on Applied Biosystems 7500 (the ABI7500). The reaction conditions are as follow: predenature at 95°C for 10 min, 40 cycles of denature at 95°C for 10 s, annealing at 60°C for 20 s and extension at 72°C for 34 s. The primers for RT‐PCR are synthesized GENEWIZ, Inc. and listed in Table 1. GAPDH was used as the internal control. The expressions of E‐cadherin, N‐cadherin, Vimentin, and Snail‐1 were calculated using ΔΔCt = [Ct_(target gene)_−Ct_(internal control)_]_experimental group_ − [Ct_(target gene)_−Ct_(internal control)_]_control group_.^21^

**Table 1:** Primer and sequences for reverse transcript polymerase chain reaction

Name of primer	Sequences (5′–3′)
E‐cadherin‐F	CGTCGAGCTCTTGACCGAAA
E‐cadherin‐R	TCAAACACCTCCTGTCCTCT
N‐cadherin‐F	AGGGGAGAGGTGCTCTACTG
N‐cadherin‐R	GGGGTAATCCACACCACCTG
Vimentin‐F	TCCGCACATTCGAGCAAAGA
Vimentin‐R	TGAGGGCTCCTAGCGGTTTA
Snail‐1‐F	CGAGCCATAGAACTAAAGCC
Snail‐1‐R	TGAGGGAGGTAGGGAAGTG
GAPDH‐F	ACCACAGTCCATGCCATCAC
GAPDH‐R	TCCACCACCCTGTTGCTGTA

Abbreviations: F, forward; R, reverse.

### 2.11 Western blot

After corresponding treatment for 48 h, cells were washed with precold PBS before cell lysis in a 100 μl/50 ml culture flask on ice for 30 min. The cell lysis was then centrifuged at 12,000 rpm for 10 min at 4°C. The supernatant was subpackaged into 0.5 ml tubes for preservation at −20°C or for protein quantification using BCA kit (Beyotime). The proteins were denatured with 6 × Sodium dodecyl sulfate (SDS) loading buffer at 100°C and separated by SDS electrophoresis. The separated proteins were transferred into membrane with 4°C precold transfer buffer for 1.5 h before blocking with tirs buffer salt +tween (TBST) prepared 5% skim milk powder for 1 h. After that, the membranes were incubated with TBST prepared primary antibodies of E‐cadherin (1:100; 14472S, Cell Signaling Technology), N‐cadherin (1:1000; 13116S; Cell Signaling Technology), Vimentin (1:1000; 5741S; Cell Signaling Technology), TRAIL (1:1000; 3219S; Cell Signaling Technology), Snail‐1 (1:500; ab53519; Abcam), Bcl‐2 (1:1000; ab182858, Abcam), Bax (1:2000; ab32503; Abcam) and β‐actin (1:1000; ab4970s; Abcam) for 4 h overnight. Then the membranes were washed with TBST for 3 × 10 min before incubation with goat anti rabbit or goat anti rat IgG (1:5000; Beijing ComWin Biotech Co, Ltd) for 2 h. After TBST wash, the protein expression of target gene was developed and observed.

### 2.12 Nude mouse models bearing Huh7 HCC xenografts and with systematic metastasis

Male NOD/SCID nude mice (*n* = 24, aged 4 weeks, 15–20 g) provided by Department of experimental animals of Kunming Medical University were used for model establishment. HCC cells, Huh7 in logarithmic phase were digested with 0.25% pancreatic enzymes before PBS washing for later centrifugation at 1000 rpm. The cell viability was determined using trypan blue staining. The viable cell number of Huh7 cells counted by a thrombocytometer was above 95%. PBS was used to adjust the concentration of HCC cell suspension into 1 × 10^7^ cells/ml. The nude mice bearing HCC xenografts (*n* = 12) were established by subcutaneous injection of HCC cell suspension (100 μl) on the left side of the abdomen, while the nude mice with systematic metastasis (*n* = 12) were established by tail intravenous injection of GFP labeled HCC cell suspension (100 μl). The implanted tumor growth was observed. When the tumor diameter reached 0.5 cm, intervention was performed to grouped the nude mice into Saline group (*n* = 4, tail intravenous injection of 100 μl of normal saline), ADSCs group (*n* = 4, tail intravenous injection of 1 × 10^7^ ADSCs) and sTRAIL‐ADSCs group (*n* = 4, tail intravenous injection of 1 × 10^7^ sTRAIL‐ADSCs). All operations in this experiment are consistent with the regulation of ethics commitment of our hospital. All efforts have been made to minimum the pain and suffering of the animals.

### 2.13 Tumor growth

The tumor growth in each group was monitored and recorded for every 3 days. Tumor volume (*V*) = *L* × *I* ^2^ × 0.52 mm^3^, in which *L* is the maximum diameter of tumor, *I* the minimum diameter of tumor. The mice were killed 21 days after model establishment to collect the tumor tissues.

### 2.14 Hematoxylin and eosin staining

Tumor tissues from nude mice were fixed in 4% paraformaldehyde (Thermo Fisher Scientific) for 48 h before being washed in running water and dehydrated with up‐graded ethanol (70%, 80%, 95% I and II, 100% I and II ethanol, each for 1 h). Then the tissues were subjected to vitrification by dimethylbenzene I and II, each for 45 min, and then immersed in paraffin I, II, and III, each for 1 h. After that, the tissues were embedded in microtome, with the slide thickness of 5 μm.

The slices were first rinsed with distill water and then stained with hematoxylin and eosin (H&E; Beijing Solarbio Science & Technology Co, Ltd) for 5 min before running water washing. Then the slices were differentiated using 1% acid alcohol for 10 s and rinsed with running water till cell nucleus are in blue. After that, the up‐graded ethanol (70%, 80%, 90%, and 95%) was used for dehydration, each for 10 min, and then absolute ethanol was applied for dehydration for twice, each for 20 min. The dehydrated slices were subjected to vitrification by dimethylbenzene for twice, each for 20 min before sealing by neutral resins.

### 2.15 Immunohistochemistry

The sections were first subjected to EDTA antigen repairing buffer (pH = 9.0) for 10 min and washed with PBS for three times, followed by incubation with 3% hydrogen peroxide solution for 10 min and PBS wash for another three times. After that, the sections were blocked with serum for 30 min and the probed with 50 µl of blocking buffer prepared primary antibody of Ki67 (1:400; 9449S; Cell Signaling Technology) for overnight at 4°C. After PBS washing away the excessive reaction buffer, the secondary antibody was added at room temperature for incubation of 0.5 h. PBS wash for three times before DAB development, nucleus staining with hematoxylin for 3 min and differentiation with 1% hydrochloric acid alcohol for 1–3 s. Additionally, the sections were washed in running water and subjected to 0.6% ammonium hydroxide treatment, dehydreation, vitrification by dimethylbenzene and mounting. Optical microscope was applied for observation and photographing.

### 2.16 In vivo image

In vivo imaging system was applied to detect the expression of GFP. Mice were anaesthetized with 35 mg/kg pentobarbital sodium, on parallel, 15 mg/ml luciferin potassium salt was injected into each mouse based on the criteria of 10 μl/g. About 5 min later injection, the tumor growth in vivo can be imaged

### 2.17 Statistical analysis

SPSS 18.0 (IBM Corp, Armonk) and GraphPad Prism 6.0 (GraphPad Software Inc) were used for data analysis. Measurement data were expressed as mean ± *SD*. Comparison between two groups was analyzed using *t* test while comparisons among groups were achieved through One‐way analysis of variance. The *p* value of less than .05 was considered to have significant difference.

## 3 RESULTS

### 3.1 Identification of ADSCs

**Figure 1:**
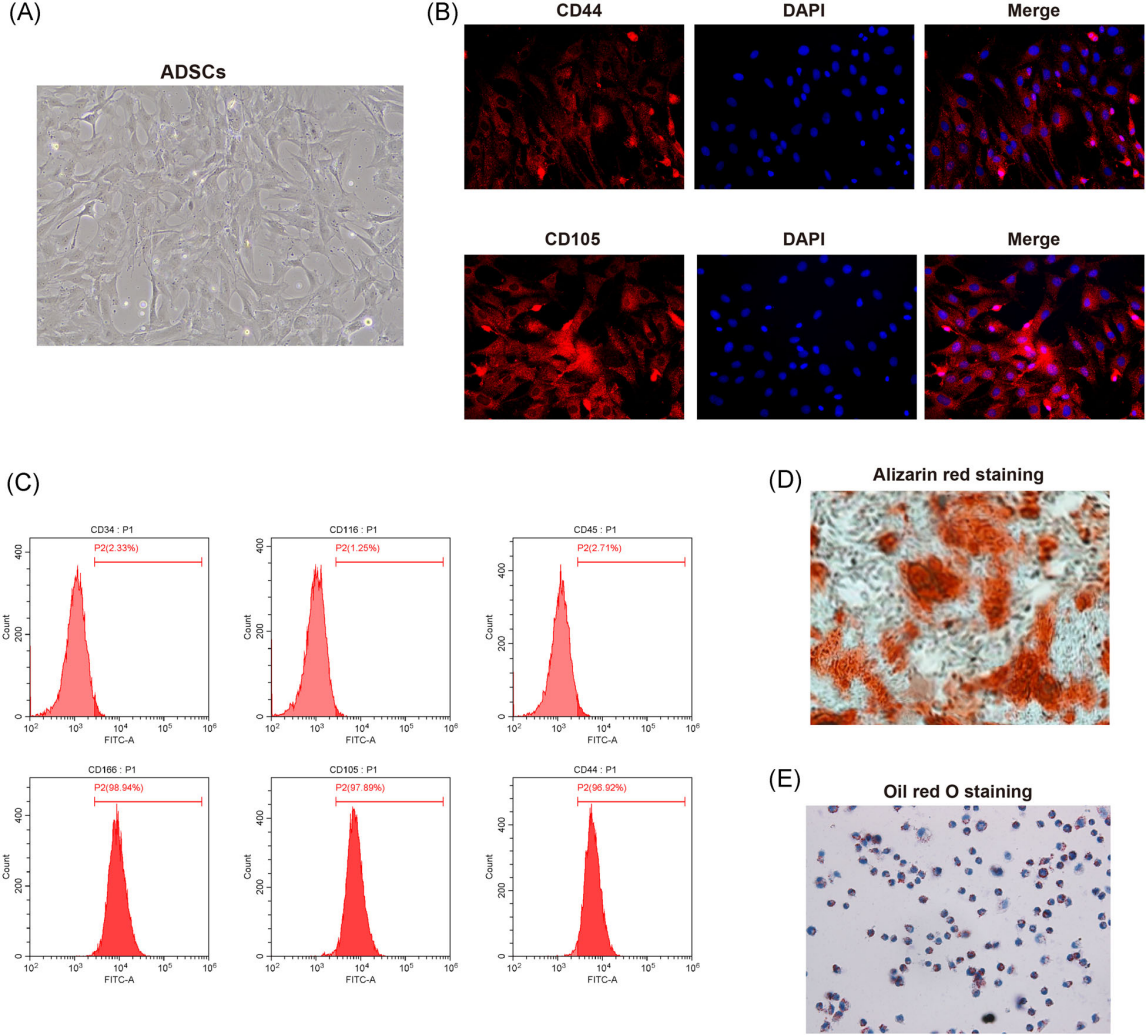
Culture, isolation and identification of ADSCs. (A), cell morphologies under microscope after isolated cells being culture for respectively 24 h (left) and 14 days (right); (B), detection of biomarkers of ADSCs, CD44 (left) and CD105 (right) using immunofluorescent staining; (C), FCM measured the expressions of CD105, CD166, CD44, CD116, CD34, and CD45 in the 4th generation of ADSCs; (D), osteogenesis differentiation potential of ADSCs was verified using alizarin red staining; (E), adipogenic differentiation potential of ADSCs was verified using oil red O staining; ADSCs, adipose‐derived stem cells; FCM, flow cytometry

After cell culture for 24 h, small amount of fusiform ADSCs are adherent to the culture flask, while after culture for 14 days, cell growth covered more than 80% area of the flask bottom. Cells were grown into colonies of fusiform or polygonal shape (Figure 1A). Immunofluorescence staining found the positive expressions of CD44 and CD105 in ADSCs (Figure 1B). FCM found that CD105, CD166, and CD44 were positively expressed while CD116, CD34, and CD45 were negatively expressed in the 4th generation of ADSCs (Figure 1C). Those observations supported the fact the isolated cells are ADSCs instead hematopoietic stem cells.

To verify the differentiation potential of ADSCs, we used alizarin red staining to see whether the ADSCs have calcium salt deposition ability. As showed in Figure 1D, observation under microscope showed the presence of large amount of red calcium salt deposits in ADSCs, suggesting the differentiation tendency toward osteogenesis. Oil red O staining found red adipose granules deposited within the cells (Figure 1E), suggesting the potential of ADSCs towards adipocyte differentiation. Above results demonstrated the isolated ADSCs possess multiply differentiation potentials, and therefore the isolated cells are adult ADSCs.

### 3.2 TRAIL modified ADSCs migrate toward HCC cells

**Figure 2:**
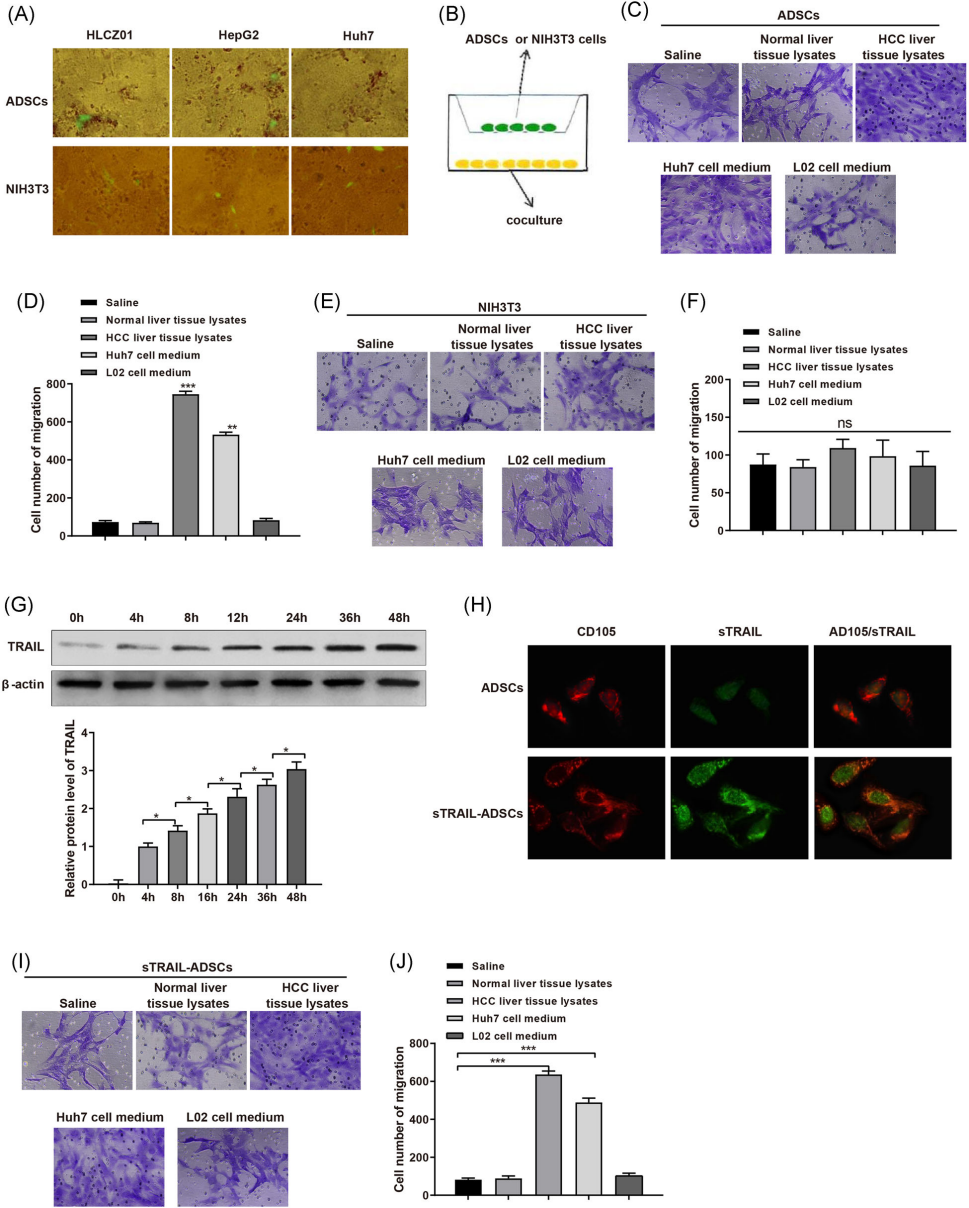
Orientated migration of sTRAIL‐ADSCs toward HCC cells. (A), AAV‐GFP labeled ADSCs or NIH3T3 were cultured with HCC cells for observation of fluorescent under fluorescence microscope; (B), the pattern of Transwell coculture system to induce migration of ADSCs or NIH3T3 cells toward normal liver histolysate, HCC histolysate, Huh7 culture medium or L02 culture medium; (C and D), effect of normal liver histolysate, HCC histolysate, Huh7 culture medium and L02 culture medium on migration of ADSCs; (E and F), effect of normal liver histolysate, HCC histolysate, Huh7 culture medium and L02 culture medium on the migration of NIH3T3; (G), Western blot detecting the expression of TRAIL in ADSCs after AAV‐CMV‐sTRAIL transfection; (H), expressions of TRAIL and CD105 in ADSCs or sTRAIL‐ADSCs; (I and J), effect of normal liver histolysate, HCC histolysate, Huh7 culture medium, and L02 culture medium on migration of sTRAIL‐ADSCs.*p < .05, ***p < .001. ADSCs, adipose‐derived stem cells; FCM, flow cytometry; HCC, hepatocellular carcinoma; TRAIL, tumor necrosis factor‐related apoptosis‐inducing ligand

To ascertain the effect of TRAIL modified ADSCs on HCC cells, we first have to make sure that ADSCs can migrate towards HCC cells. To verify our hypothesis, HCC cell lines, HLCZ01, HepG2, and Huh7 cells were cultured for 2 days before cocultured with AAV‐GFP labeled ADSCs for 2 days. Fluorescence microscope found gathered green fluorescent in HLCZ01, HepG2, and Huh7 cells, while those fluorescent was dispersedly observed in AAV‐GFP labeled NIH3T3 cells (mouse embryonic fibroblasts; Figure 2A).

To better understand the migration pattern of ADSCs towards HCC cells, normal liver histolysate, HCC histolysate, Huh7 culture medium or L02 culture medium are place in Transwell coculture system to induce cell migration (Figure 2B). The Transwell system found either normal liver histolysate or L02 culture medium can induce the migration of ADSCs, while ADSCs were migrated towards HCC histolysate and Huh7 culture medium, among which HCC histolysate had better migration induce ability than Huh7 culture medium (Figure 2C,D, *p* < .01). Nevertheless, no migration of NIH3T3 cells was observed (Figure 2E,F, *p* > .05). Those results hinted that HCC cells may release or express certain chemokines to induce the migration of ADSCs. No migration induce ability was found in NIH3T3 cells, therefore the migration of ADSCs towards HCC cells may accredit to certain chemokine receptors.

TRAIL is a member of the TNF family with the specific ability to induce apoptosis of tumor cells. ADSCs were transfected with stably expressed sTRAIL through AAV‐CMV‐sTRAIL. Western blot detected TRAIL expression in culture medium of AAV infected ADSCs with TRAIL expression level in time depend manner (Figure 2G, *p* < .01). No TRAIL expression was detected in culture medium of ADSCs without AAV‐CMV‐sTRAIL transfection (Figure 2G). We also found positive expression of sTRAIL in AAV‐CMV‐sTRAIL transfected ADSCs, in which CD105 was positively expressed (Figure 2H). Taken together, secreted sTRAIL was stably expressed in ADSCs, and AAV‐CMV‐sTRAIL transfection had minimum biological effect on ADSCs.

The migration assay in Transwell assay showed that normal liver histolysate and L02 culture medium failed to induce the migration of either ADSCs or sTRAIL‐ADSCs. Meanwhile, both ADSCs and sTRAIL‐ADSCs can migrate towards HCC histolysate and Huh7 culture medium (Figure 2I,J, *p* < .01). Those results showed that ADSCs with stably expression of secreted sTRAIL still can migrate toward HCC cells.

### 3.3 TRAIL modified ADSCs induce apoptosis of HCC cells

**Figure 3:**
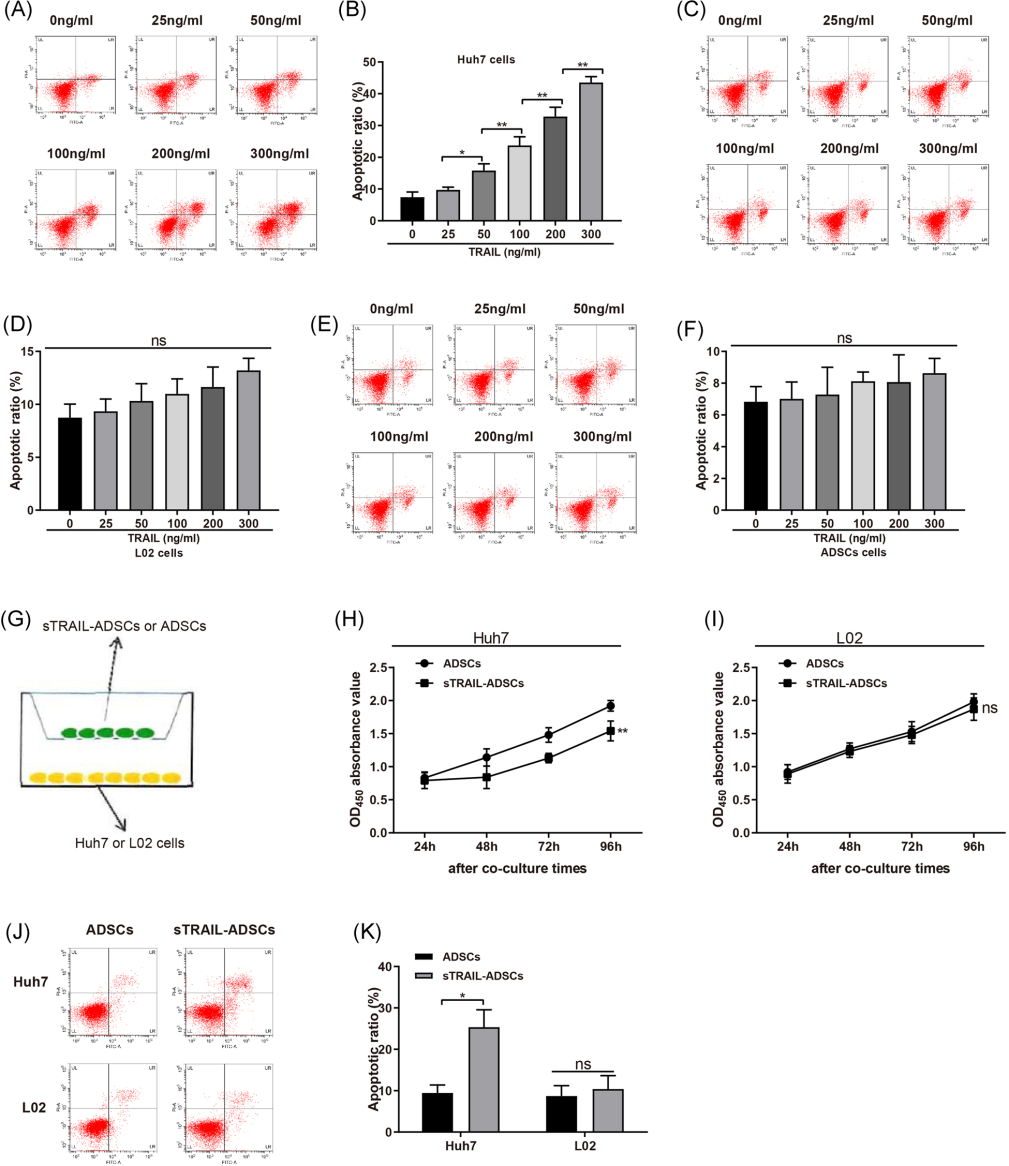
Coculture with sTRAIL‐ADSCs could induce cell apoptosis of Huh7 cells. (A and B), FCM to measure the effect of sTRAIL on apoptosis of Huh7 cells; (C and D), FCM to measure the effect of sTRAIL on apoptosis of L02 cells; (E and F), FCM to measure the effect of sTRAIL on apoptosis of ADSCs; (G), coculture system of sTRAIL‐ADSCs or ADSCs with Huh7 or L02 cells; (H), CCK‐8 assay to detect the effect of sTRAIL‐ADSCs or ADSCs to proliferation of Huh7 cells; (I), CCK‐8 assay to detect the effect of sTRAIL‐ADSCs or ADSCs to proliferation of L02 cells; (J and K), FCM to measure the effect of coculture of sTRAIL‐ADSCs or ADSCs with Huh7 or L02 cells on apoptosis of Huh7 or L02 cells. ns: *p* > .05, *p < .05, **p < .01. ADSCs, adipose‐derived stem cells; CCK‐8, Cell Counting Kit‐8; FCM, flow cytometry; HCC, hepatocellular carcinoma; TRAIL, tumor necrosis factor‐related apoptosis‐inducing ligand

Huh7, L02 cells and ADSCs were cultured in sTRAIL culture medium (concentrations of 0, 25, 50, 100, 200, and 300 ng/ml) for 24 h before FCM was applied to measure cell apoptosis. The results showed that incubation with sTRAIL (even at the concentration of 300 ng/ml) had no remarkable effect on apoptosis rate of ADSCs and L02 cells. Meanwhile, the apoptosis rate of Huh7 cells after incubation with sTRAIL (concentration of more than 50 ng/ml) was obviously higher than that without sTRAIL (Figure 3A,B, *p* < .01) in a dose dependent manner. Those observations indicate that sTRAIL can induce the apoptosis of Huh7 cells, but fail to affect L02 cells (Figure 3C,D) and ADSCs (Figure 3E,F).

To further explore the effect of sTRAIL‐ADSCs on HCC cells, sTRAIL‐ADSCs or ADSCs were cocultured with Huh7 cells or L02 cells in a Transwell system (Figure 3G). CCK‐8 assay on cell proliferation ability detected that compared with coculture with ADSCs, sTRAIL‐ADSCs coculture can substantially suppress the proliferation ability of Huh7 cells (*p* < .01), but not L02 (Figure 3H,I). Consistently, FCM revealed that sTRAIL‐ADSCs coculture can enhance cell apoptosis of Huh7 cells, compared with coculture with ADSCs (Figure 3J,K, *p* < .01). No significant difference was found in L02 cells. Collectively, sTRAIL‐ADSCs can promote apoptosis of Huh7 cells, while sTRAIL‐ADSCs had no toxicity on L02 cells.

### 3.4 TRAIL modified ADSCs may suppress subcutaneously implanted tumor in nude mouse

**Figure 4:**
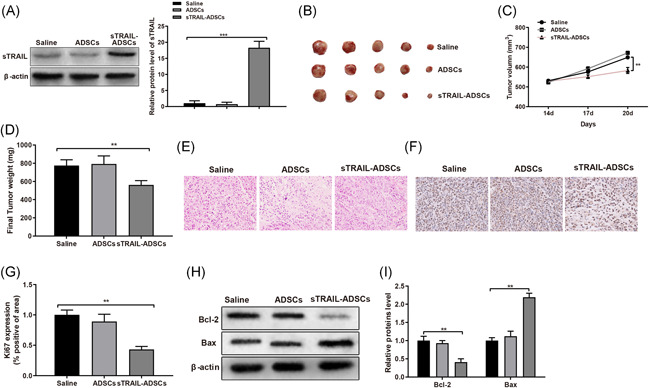
sTRAIL‐ADSCs can suppress the growth of subcutaneously implanted HCC in nude mouse. (A), sTRAIL expression in each group was measured by Western blot; (B), tumor size in nude mice of each group; (C), comparison on tumor size; (D), tumor weight; (E), H&E staining for tumor tissues (×400); (F and G), immumohistochemical staining for Ki67 detection (×400); (H and I), Western blot for detection on apoptotic protein Bcl‐2 and Bax. **p < .01, ***p < .001. ADSCs, adipose‐derived stem cells; CCK‐8, Cell Counting Kit‐8; FCM, flow cytometry; HCC, hepatocellular carcinoma; H&E, hematoxylin and eosin; TRAIL, tumor necrosis factor‐related apoptosis‐inducing ligand

As showed above that sTRAIL‐ADSCs can induce cell apoptosis of HCC cells, it would be intriguing to explore whether sTRAIL‐ADSCs can be applied for HCC therapy. Huh7 cells were subcutaneously injected into nude mice. On parallel, mice injected with ADSCs or normal saline were used as control groups. Detection by Western blot showed that sTRAIL was highly expressed in sTRAIL‐ADSCs group, while nearly no sTRAIL expression was detected in ADSCs group and Saline group (Figure 4A, *p* < .01). Therefore, sTRAIL‐ADSCs can increase sTRAIL expression in tumor tissues of nude mouse.

Measurement on tumor size and tumor weight found that mice injected with ADSCs had slightly increased tumor size and weight in comparison to that in Saline group, but no statistically significant difference was detected. In contrast to tumor size and weight measured in Saline group, those data were decreased in mice in sTRAIL‐ADSCs group (Figure 4B,C, *p* < .01). H&E staining on tumor slice showed that tumor cells in sTRAIL‐ADSCs group had vague cell cores with dense chromatin, even with fragmented nucleus, swollen or balloon shaped cytoplasm. The observation on cells in ADSCs group and Saline group showed that cell nucleuses are regularly arranged without fragments or cytoplasm changes (Figure 4E). Immumohistochemical staining was applied to detect the expression of Ki67. The results showed that compared with Saline group, the Ki67 was slightly increased in ADSCs group, although no significant difference was detected, while the Ki67 in sTRAIL‐ADSCs was remarkably decreased (Figure 4F,G, *p* < .01). Western blot detection on apoptotic‐related protein Bax and Bcl‐2 showed the expression levels of Bax and Bcl‐2 in ADSCs were slightly altered compared with those in Saline group, but failing to achieve any significant difference. The further comparison showed that the Bcl‐2 in sTRAIL‐ADSCs group was decreased, while Bax was elevated in comparison to that in Saline group (Figure 4H,I, *p* < .01). Collectively, TRAIL modified ADSCs may be able to achieve certain therapeutic efficiency for subcutaneously implanted HCC.

### 3.5 TRAIL modified ADSCs suppress micrometastasis of HCC

**Figure 5:**
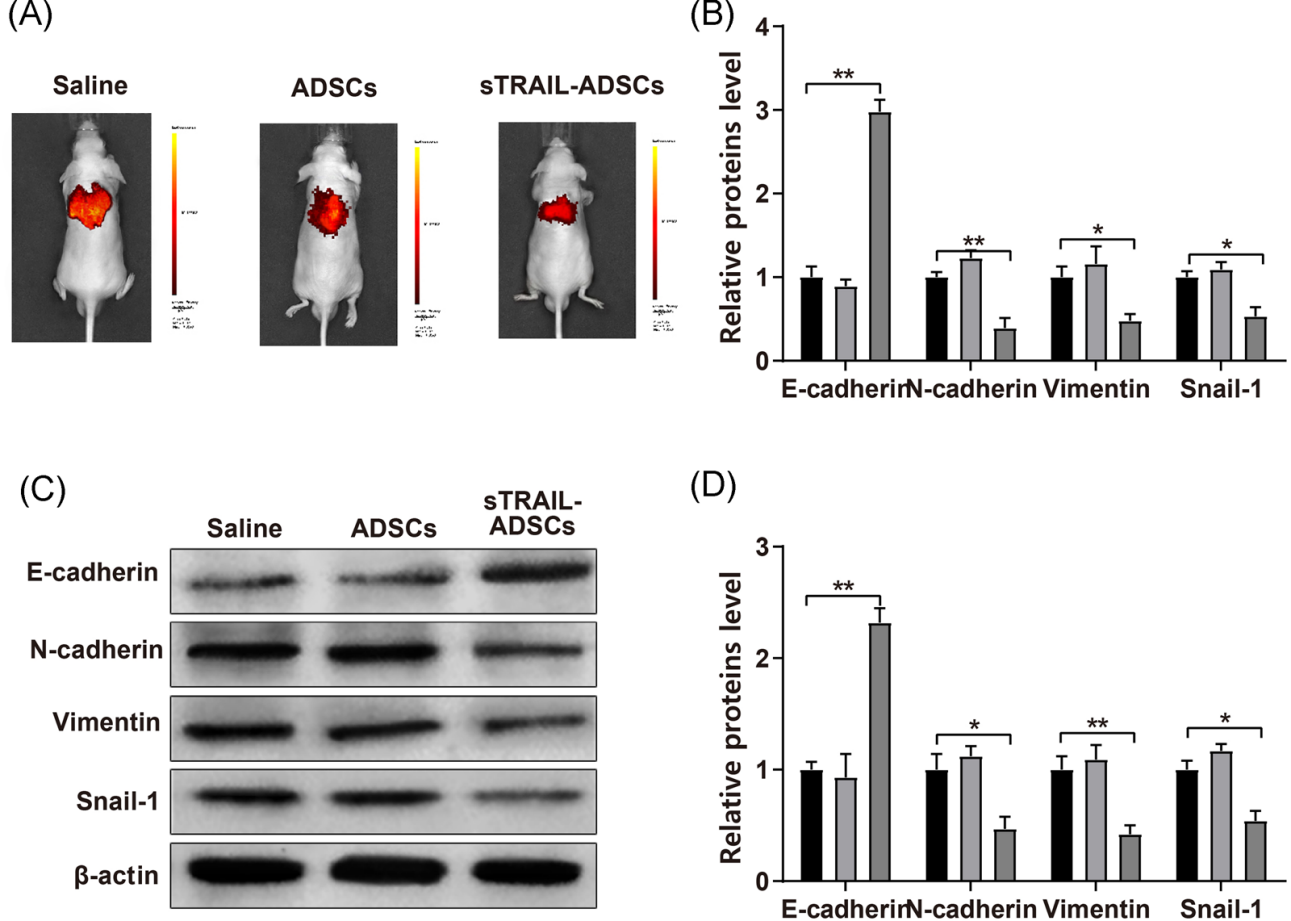
Suppressive effect of sTRAIL‐ADSCs on micrometastasis of HCC in nude mouse. (A), distribution of micrometastasis in nude mouse was monitored by in vivo image system; (B), PCR was applied to detect the mRNA expressions of EMT related biomarkers (E‐cadherin, N‐cadherin, Vimentin, and Snail‐1); (C and D), Western blot was applied to detect the mRNA expressions of EMT related biomarkers (E‐cadherin, N‐cadherin, Vimentin, and Snail‐1). *p < .05, **p < .01. ADSCs, adipose‐derived stem cells; EMT, epithelial‐mesenchymal transition; HCC, hepatocellular carcinoma; mRNA, messenger RNA; TRAIL, tumor necrosis factor‐related apoptosis‐inducing ligand

GFP labeled Huh7 was injected into nude mice through tail vein injection for establishment of HCC metastasis model. In vivo image system was applied to evaluate the effect of sTRAIL‐ADSCs on tumor metastasis. The images presented that micrometastasis in sTRAIL‐ADSCs group was substantially decreased when compared with Saline group (Figure 5A). Similar micrometastasis condition was displayed in Saline group and ADSCs group. The expressions of epithelial‐mesenchymal transition biomarkers (E‐cadherin, N‐cadherin, Vimentin, Snail‐1) were also measured among the three groups using quantitative PCR and Western blot. No noticeable difference was found between Saline group and ADSCs group. Nevertheless, E‐cadherin was elevated, while N‐cadherin, Vimentin and Snail‐1 were decreased in sTRAIL‐ADSCs group in comparison to that in Saline group (Figure 5B,D, *p* < .01). Collectively, TRAIL modified ADSCs can inhibit the micrometastasis of HCC in nude mouse.

## 4 DISCUSSION

Therapy for HCC patients often met with failure, relapse and metastasis since majority of patients are initially diagnosed at the advanced stage.^22^ ADSCs are gradually accepted as a new breakthrough for cancer treatment considering their potential of serving as an available cell delivery carrier. The multidifferentiation potential enables ADSCs to differentiate into natural killer cells to exert its antitumor effect,^23^ or urothelium and smooth muscle cells for ureter reconstruction.^24^ Meanwhile, secretions of ADSCs are reported to enhance tumor cell growth, as in certain amount of clinical studies, ADSCs were believed to facilitate tumor growth by providing a suitable microenvironment for tumor cells.^25^, ^26^, ^27^ We extracted ADSCs from human adipose tissues with the aim to explore the possible effect of TRAIL modified ADSCs on HCC development both in vivo and in vitro. Evidence in this study denied the tumor suppressive role of ADSCs in HCC cells, however, TRAIL modified ADSCs were showed to inhibit tumor proliferation and metastasis of HCC cells both in vivo and in vitro.

Firstly ADSCs were extracted from human adipose tissues and confirmed through immunofluorescence staining and FCM, in which the positive expressions of CD105, CD166, and CD44 as well as negative expressions of CD116, CD34, and CD45 were found. Heametopoietic stem cell markers such as CD34 and CD45^28^ are negatively expressed in ADSCs, indicating no heametopoietic stem cells existed in the extracted ADSCs. Staining on ADSCs also supported the multipotent differentiation potentials of the extracted cells. In addition to that, ADSCs were also found to migrate toward HCC cells in a Transwell coculture system, but barely towards the normal liver histolysate, Huh7 culture medium or L02 culture medium. A possible explanation was proposed that chemokines secreted by tumor cells were abundantly expressed in tumor site, which in turn may induce the migration of ADSCs.^29^ But the detailed mechanism for ADSCs migration remains to be determined.

To modify the ADSCs, we extracted and transfected with AAV‐CMV‐sTRAIL into ADSCs. The detection on TRAIL indicates that TRAIL modification would not affect the biological function of ADSCs. Further results also supported that TRAIL modified ADSCs also possess the multipotent differentiation ability of ADSCs. TRAIL is one of the wildly used therapeutic agents which can specifically target tumor cells without affecting the normal cells.^9^ Little side effect of TRAIL‐based therapy has prompted the application of recombinant human TRAIL vector in cancer treatment as TRAIL had minimum effect on activation of oncogenic nuclear factor kappa B pathway.^9^ Our results in this study showed that TRAIL modified ADSCs can induce cell apoptosis in vitro. Further experiments in vivo supported the tumor suppressive role of TRAIL modified ADSCs in xenografted nude mouse. The cell apoptosis in tumor cells may achieved by either an intrinsic way or an extrinsic way, among which the latter approach was initiated by the binding of extracellular ligands with transmembrane receptors, consequently leading to caspase activation and cell death.^30^ TRAIL induced cell apoptosis through activating death receptor (DR) 4 or/and DR5 in an extrinsic way, which in turn activates a caspase cascade to execute cell apoptosis.^31^ The sequential signaling events involve the activation of DRs including Fas, TRAIL‐receptor, which leads to the formation of a death‐inducing signaling complex (DISC), composed of Fas‐associated death and pro‐caspase 8/10. Then the DISC is processed and released into cytoplasm to initiate effector caspase (caspase 3 or caspase 7) mediated apoptosis.^32^ Similar results were reported in melanoma, which showed that TRAIL‐ADSCs could facilitate cell apoptosis of melanoma cells in a dose‐dependent manner.^33^ The application of ADSCs as a carrier in liver disease showed that ADSCs is able to transfers miR‐181‐5p to lesions.^34^

Taken together, evidence collected in this study demonstrated that TRAIL modified ADSCs exhibited the characteristics of ADSCs, such as migrating toward HCC cells, multipotent differentiation ability. Meanwhile, TRAIL modified ADSCs can also induce cell apoptosis of HCC cells and obstruct tumor growth in xenografted nude mouse. Therefore, TRAIL modified ADSCs are potential therapeutic approaches for HCC treatment and worth widely application in clinical stage given that more rigorous and well‐designed studies confirmed the effect of TRAIL modified ADSCs on HCC therapy.

## CONFLICT OF INTERESTS

The authors declare that there are no conflict of interests.

## AUTHOR CONTRIBUTIONS

Zhuo Liu and Shaojie Li conceived the ideas. Zhuo Liu and Shaojie Li designed the experiments. Min Tang, Jian Zeng, and Xin Zhou performed the experiments. Huanyu Li and Tiexiang Ma analyzed the data. Xiang Liu, Feng Li, and Bin Jiang provided critical materials. Zhuo Liu, Min Tang, and Ming Zhao wrote the manuscript. Shaojie Li and Ying Chen supervised the study. All the authors have read and approved the final version for publication.

## Data Availability

The datasets used or analyzed during the current study are available from the corresponding author on reasonable request.
